# Therapeutic Advances in Regulating the Hepcidin/Ferroportin Axis

**DOI:** 10.3390/ph12040170

**Published:** 2019-11-25

**Authors:** Zachary J. Hawula, Daniel F. Wallace, V. Nathan Subramaniam, Gautam Rishi

**Affiliations:** 1 Institute of Health and Biomedical Innovation, Queensland University of Technology (QUT), Brisbane, Queensland 4059, Australia; zachary.hawula@hdr.qut.edu.au (Z.J.H.); d5.wallace@qut.edu.au (D.F.W.); 2 School of Biomedical Sciences, Queensland University of Technology (QUT), Brisbane, Queensland 4059, Australia

**Keywords:** iron metabolism, hepcidin, iron homeostasis, ferroportin

## Abstract

The interaction between hepcidin and ferroportin is the key mechanism involved in regulation of systemic iron homeostasis. This axis can be affected by multiple stimuli including plasma iron levels, inflammation and erythropoietic demand. Genetic defects or prolonged inflammatory stimuli results in dysregulation of this axis, which can lead to several disorders including hereditary hemochromatosis and anaemia of chronic disease. An imbalance in iron homeostasis is increasingly being associated with worse disease outcomes in many clinical conditions including multiple cancers and neurological disorders. Currently, there are limited treatment options for regulating iron levels in patients and thus significant efforts are being made to uncover approaches to regulate hepcidin and ferroportin expression. These approaches either target these molecules directly or regulatory steps which mediate hepcidin or ferroportin expression. This review examines the current status of hepcidin and ferroportin agonists and antagonists, as well as inducers and inhibitors of these proteins and their regulatory pathways.

## 1. Introduction

Iron is an essential component of many cellular processes including oxygen transport, DNA replication and repair and as an enzyme cofactor [1,2]. While iron is abundant within the Earth’s crust, ferrous (Fe^2+^) iron spontaneously reacts with oxygen to form ferric (Fe^3+^) iron, which is insoluble under physiological conditions [3]. This presents the first of many challenges that organisms face in meeting their needs for adequate iron absorption, as organisms require mechanisms to convert Fe^3+^ to Fe^2+^. The second challenge organisms face is iron’s ability to undergo redox reactions via Fenton chemistry, which result in reactive oxygen species (ROS). These destructive ROS have the potential to damage proteins, lipid membranes and nucleic acids and result in tissue damage [3,4]. As there is currently no known export mechanism for excess iron, its metabolism is tightly regulated through transcriptional, post-transcriptional, translational, and post-translational mechanisms to prevent toxic iron excess while maintaining the metabolic needs of the organism [5,6].

An adult human body contains 3–5 g of iron, much of which is supplied to developing erythroblasts for incorporation within the haem group of haemoglobin (70% of bodily iron) [3]. Other tissues which are major users of iron include the muscles (2–3%) where it is present in the haem group of myoglobin, tissue macrophages (5%) involved in red blood cell (RBC) recycling and liver hepatocytes (20%) where excess iron is stored within ferritin. Humans require around 20–25 mg of iron per day for the synthesis of new RBCs; however, only about 1–2 mg of iron is absorbed per day via the enterocytes of the duodenum. The majority of iron (90%) is supplied by the recycling of RBCs by macrophages [2,6].

## 2. Hepcidin Regulation

Hepcidin is an 84-amino-acid long prepropeptide produced in hepatocytes and excreted in urine as a 25-amino-acid mature form [7]. It was originally thought to function solely as an antimicrobial peptide as it is upregulated under inflammatory conditions and as such is considered to be a type two acute-phase reactant due to its regulation via interleukin 6 (IL-6) [7,8,9]. The 24-amino-acid N-terminal signal peptide of pre-prohepcidin is cleaved to produce prohepcidin, comprising 60 amino acids [10]. The mature 25-amino-acid form of hepcidin [7] is generated via cleavage of the pro-region by furin-like prohormone convertases [10]. The carboxy terminus of the mature hepcidin, comprising four highly conserved disulphide bonds, forms a β-hairpin; the N-terminus is unstructured and essential for ferroportin (FPN) interaction [11]. This interaction results in reduced membrane concentrations of FPN, thus lowering levels iron availability to tissues [12].

**Figure 1:**
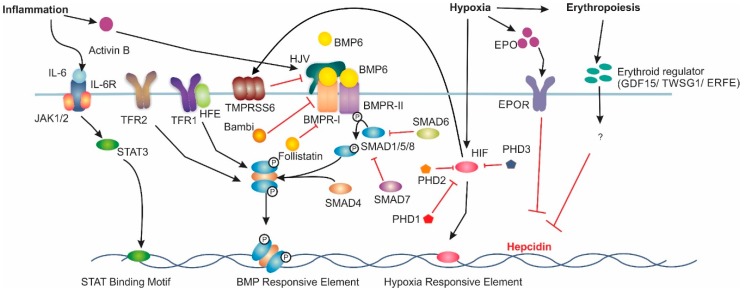
Major Signalling Pathways Involved in Hepcidin Regulation. Increased BMP6 levels induce hepcidin mRNA expression via the bone morphogenic protein (BMP)/sma and mothers against decapentaplegic homologue (SMAD) pathway. Under inflammatory conditions, interleukin 6 (IL-6) induces hepcidin mRNA via the Jak/signal transducer and activator of transcription (STAT) pathway. Hypoxia induces hepcidin downregulation through increases in erythropoietin (EPO) expression. Hypoxia Inducible Factor (HIF) can also directly downregulate hepcidin. Additionally, HIF upregulates matriptase-2 expression, which results in decreased hepcidin expression. Lastly, erythropoiesis has been theorised to reduce hepcidin expression acting through growth differentiation factor 15 (GDF-15), twisted gastrulation factor 1 (TWSG1) or erythroferrone (ERFE), however the exact mechanism is not understood. Interleukin-6 Receptor (IL6-R), Transferrin Receptor 1 (TFR1), Hemojuvelin (HJV), Bone Morphogenic Protein Receptor Type 1 (BMPR-I), Bone Morphogenic Protein Receptor Type 2 (BMPR-II), Erythropoietin Receptor (EPOR) and prolyl hydroxylase domain containing enzymes (PHD) are involved in hepcidin regulation.

The interplay between the iron exporter FPN and the hepatic peptide hormone, hepcidin, is central to iron homeostasis. Hepcidin binds to membrane-bound FPN and induces its internalisation and eventual degradation. The expression of these two proteins is highly regulated. The main regulatory pathway for liver hepcidin expression is the bone morphogenetic protein/Sma mothers against decapentaplegic (BMP/SMAD) pathway (Figure 1). The ligand BMP6, believed to be produced by liver sinusoidal endothelial cells [13], has been found to be the primary BMP associated with hepcidin transcription [14,15]. However, BMPs 2, 4 and 9 have also been shown to increase hepcidin expression [16,17]. Increased body iron stores induce BMP6 expression in the liver; BMP6 then binds to membrane-bound BMP receptors (BMPRs) with hemojuvelin (HJV) acting as a co-receptor [18]. BMPRs type 1 (activin-like kinase (ALK) 2 and ALK3) and type 2 (BMPR2 and ActR2A) are critical for iron balance [19,20]. BMP6 binding to BMPRs results in the phosphorylation of intracellular SMAD1/5/8 [21]. Phosphorylated SMAD1/5/8 then complexes with SMAD4, which translocates to the nucleus [22], where it binds to BMP-responsive elements (BMP-RE) on the hepcidin gene promoter inducing hepcidin expression [23].

An et al. demonstrated that phosphorylation of SMAD1/5/8 is regulated by the inhibitory SMADs 6 and 7 [24]. Hepatocyte-specific *Smad7* knockout mice demonstrated a decrease in non-haem iron within the liver and spleen, in addition to decreases in L-ferritin and FPN levels [24]. Conversely, hepcidin and phosphorylated SMAD1/5/8 levels were increased in these mice [24]. SMAD6, BMP, activin membrane-bound inhibitor homolog (Bambi) and follistatin have been shown to be inhibitors of hepcidin expression in a *Smad7* knockout mouse model fed an iron-rich diet [24]. SMAD6 is known to inhibit the phosphorylation of other SMAD proteins while both Bambi and follistatin inhibit the BMP pathway through interacting with the BMPRs and BMPs respectively [24]. Interestingly, An et al. found that SMAD6 and Bambi were controlled by the BMP/SMAD pathway, while follistatin was unaffected [24]. This may indicate why SMAD6 and Bambi are unable to substitute for SMAD7 under normal iron conditions.

BMP6 and iron levels have also been shown to increase the expression of the transmembrane serine protease, matriptase-2 (TMPRSS6) [25]. TMPRSS6 acts as a negative regulator of hepcidin, having been shown to cleave HJV and thus reduce the available membrane-bound HJV [26]. In addition, Lin et al. found that soluble HJV (sHJV) competes with membrane-bound HJV for ligation with BMPs resulting in hepcidin suppression [27].

Hepcidin regulation under inflammatory conditions involves the IL6/signal transducer and activator of transcription (IL6/STAT) pathway [28]. IL6 released during inflammation binds to its receptors, which in turn induce Janus kinase 1 (JAK) to phosphorylate STAT3 [29]. STAT3 translocates to the nucleus where binding to the STAT binding motif on the *Hepcidin* gene promoter activates expression [28]. Interestingly, intact SMAD1/5/8 function is required for maximal induction of hepcidin via the IL6/STAT3 pathway [30]. It has been suggested that activin B may be responsible for the cross talk between the IL6/STAT3 and BMP/SMAD pathways. Activin B promotes hepcidin activation, acting as a surrogate ligand for SMAD1/5/8 in the BMP/SMAD pathway during infection. Activin B interacts with type 2 BMPR ActR2A and type 1 receptors ALK2 and ALK3 to stimulate *hepcidin* expression via SMAD1/5/8 phosphorylation as described above [30,31].

In addition to the BMP6/SMAD and IL6/STAT pathways, iron levels are also regulated by hypoxia. Hypoxia Inducible Factor (HIFs), members of the heterodimeric nuclear transcription factor family are the main protein complexes that result in changes in gene expression under hypoxic conditions [32].

HIF complexes regulate a large variety of genes, although the current review focuses on the genes involved with iron regulation. One of the most well studied iron pathway genes regulated by HIF is erythropoietin (EPO). Initially, it was believed that HIF1α was the major HIF isoform involved with EPO regulation, however multiple knockout studies in mice have confirmed that HIF2α is the primary regulator of hypoxia induced EPO expression [33,34]. This led to the discovery of EPO-dependent mechanisms of hepcidin downregulation. Lui et al. discovered HIF suppression of hepcidin required EPO-induced erythropoiesis in a *Vhl/Epo*^-/-^ mouse model [35]. EPO independent mechanisms for HIF regulation of hepcidin have also been discovered. Peyssonnax et al. found murine and human hepcidin contains a Hypoxia-Response Element (HRE) within the hepcidin promoter that results in its down regulation [36]. The ability of HIF1α to downregulate hepcidin was shown in vivo using a *Hif-1α^flox/flox^* mouse model given an iron-deficient diet for 20 days that resulted in a 10-fold increase in hepcidin when compared with WT [36]. However, the direct role of HIF1α on human hepcidin has come into question with subsequent studies suggesting no direct role for HIF [37].

HIF1 also indirectly regulates hepcidin through proteins involved with the previously mentioned BMP6/SMAD pathway. As previously discussed TMPRSS6 cleaves HJV decreasing the levels of membrane-associated HJV which acts to reduce hepcidin production [38]. Maurer et al. discovered a HRE within the promoter region of TMPRSS6 [39]. Lakhal et al. also demonstrated that TMPRSS6 expression increased in a HIF1-dependent manner during hypoxia [40].

Erythroblasts are responsible for utilising the largest proportion of iron within the body to produce haemoglobin [41]. Previous studies have shown that stimulated erythropoiesis supresses hepcidin expression [41]; thus, it was long theorised that an erythroid regulator of hepcidin exists. However, the exact molecular mechanism for this regulation is currently unclear. Several candidate molecules have been proposed as the erythroid regulator of iron homeostasis. Growth differentiation factor 15 (GDF-15) and twisted gastrulation factor 1 (TWSG1) are both cytokines produced by erythroblasts which have been found to supress hepcidin expression in human liver cells [42,43]. However, in a G*df-15* knockout mouse, where erythropoiesis was stimulated via phlebotomy, there was no decrease in hepcidin expression [44]. Similarly, *Twsg1* was not increased in various mouse models of anaemia [45]. Currently, the most likely candidate for the erythroid regulator for hepcidin suppression is erythroferrone (*ERFE*). *Erfe* knockout mice have been shown to fail to supress hepcidin following stimulated erythropoiesis, whereas heterozygotes display an intermediate degree of suppression [46]. In addition, knockout of *Erfe* in the Hbb^(Th3/+)^ thalassemia intermedia mouse model restored proper hepcidin expression with partial protection from iron overload [46]. However, *Erfe* knockout mice displayed no significant difference from control mice, suggesting erythroferrone plays a role as a stress erythropoiesis specific regulator and not the main erythropoiesis regulator [47].

### Extrahepatic Hepcidin

In addition to the liver, hepcidin is also synthesised in a number of other organs including adipose tissue, brain kidney, heart, spleen, pancreas and stomach [48]. Within the kidney, where hepcidin is produced in the cortical thick ascending limb, hepcidin was been shown to play a role in the absorption of non-haem iron through the down regulation of both divalent metal transport 1 (DMT1) and FPN [48]. The role of hepcidin within the spleen, stomach and the brain appear to be linked with its antimicrobial ability. Several macrophage cell types have been found to synthesise hepcidin when challenged with pathogens. Sow et al. infected RAW264.7 macrophages, mouse bone marrow-derived macrophages and human THP-1 monocytic cell with Mycobacterium *sp.*, which stimulated hepcidin mRNA and protein synthesis [49]. Within the stomach, hepcidin expression is regulated by bacterial infection and involved with gastric acid production [50]. Hepcidin in the brain has been found to be upregulated via inflammatory responses [51,52]. Hepcidin synthesis in adipose tissue has also been reported; however, the exact role of this type of hepcidin remains largely unknown [48]. Lastly, hepcidin production within the pancreas has been suggested to play a role in the regulation of insulin [48]. These secondary roles of hepcidin will need to be considered when drug candidates undergo animal studies and clinical trials to ensure these critical roles of hepcidin are not altered.

## 3. Regulation of FPN

**Figure 2:**
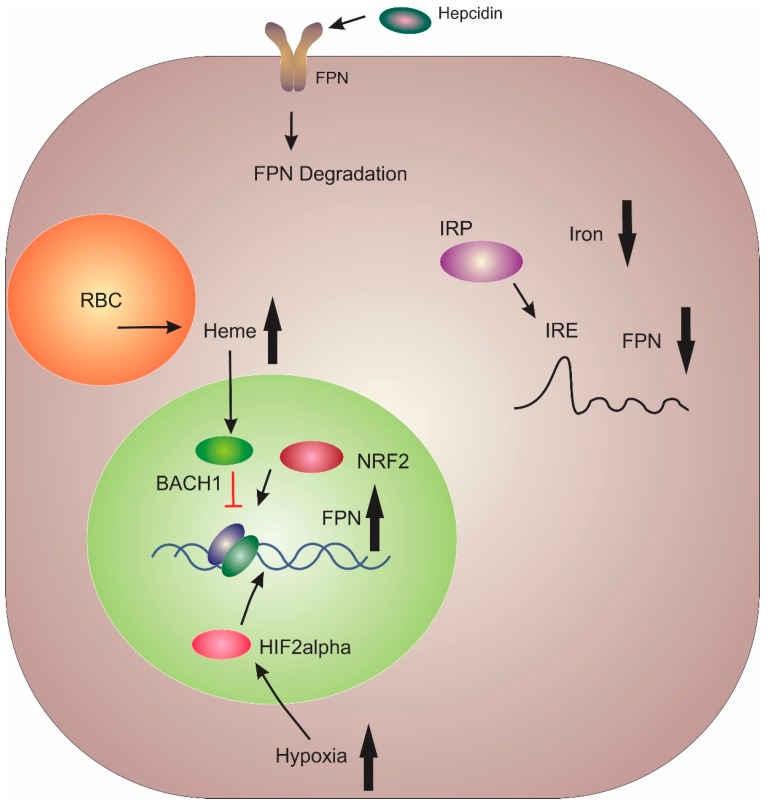
Major Regulatory mechanisms for ferroportin. BTB Domain and CNC Homolog 1 (BACH1) and Nuclear factor erythroid 2-related factor 2 (NRF2) act as a transcriptional repressor and activator of ferroportin (FPN) transcription, respectively. Cytoplasmic iron levels regulate the interaction between iron-responsive element-binding proteins (IRPs) and the iron response element (IRE) located on the 5’ end of FPN mRNA. Decreased iron levels result in IRP binding to the IRE and reduced FPN expression. Enterocytes express a variant of FPN that lacks the 5’ IRE. Hypoxia induces FPN transcription in enterocytes via hypoxia inducible factor 2α. Lastly, FPN is controlled post-translationally through complexation with hepcidin, which results in internalisation and degradation.

The sole iron exporter FPN was identified by three independent research groups in early 2000 [53,54,55]. FPN is highly expressed in the liver Kupffer cells, periportal hepatocytes, duodenal enterocytes, splenic red pulp macrophages, and the placental syncytiotrophoblast [6]. FPN typifies the multilevel regulatory pathway of iron homeostasis (Figure 2). The primary method of FPN regulation is post-translationally via hepcidin [11]. Once hepcidin has bound to FPN, it results in its ubiquitination, internalisation and degradation [56]. Thus, this interaction between hepcidin and FPN plays a central role in the regulation of bodily iron levels.

Btb and cnc homology 1 (BACH1) and nuclear factor erythroid 2-like (NRF2) proteins act as transcriptional repressors and activators, respectively, for FPN, in response to cytosolic haem levels within macrophages [57]. At the post-transcriptional level, microRNA (miRNA) miR-485-3p has been shown to inhibit FPN expression during iron deficiency [58]. Translational regulation of FPN occurs via iron-responsive element-binding proteins (IRPs) binding to the iron response element (IRE) located in the 5’ UTR of FPN mRNA [57]. This results in suppression of *FPN* mRNA translation in enterocytes and macrophages under low iron conditions [59,60]. Within the duodenum, hypoxia results in the stabilisation of HIF2α, which has been shown to increase FPN expression [6]. Additionally, a variant of FPN which lacks the 5’ UTR is predominantly expressed in enterocytes which results in sustained FPN expression in enterocytes even under iron deficiency conditions [6].

FPN expression has also been shown to be downregulated in both liver and peritoneal macrophages by lipopolysaccharide (LPS) injections [61]. This regulation of *FPN* mRNA was shown to be a result of macrophage polarisation. Wortmannin (a phosphoinositide 3-kinase inhibitor) abolished FPN deregulation in LPS-challenged macrophages while the p38-mitogen-activated protein kinase inhibitor, SB203580, intensified FPN mRNA down-regulation [61]. Toll-like receptors 2, 3 and 4 have also been found to effect FPN downregulation under inflammatory conditions. However, their mode of action is only partially understood [6]. This has led to FPN being classified as a negative acute-phase protein [62].

## 4. Hepcidin/FPN Axis Dysregulation

Dysregulation of the hepcidin– FPN axis has been associated with the development of cancer. This most likely results from higher rates of cell proliferation requiring greater demand for iron [63]. Thus, reduced hepatic hepcidin was found to offer a protective effect against the progression of lung [64] and breast cancer [65,66]. In addition, the over-expression of FPN also displayed a protective effect as FPN overexpression reduced cell division and colony formation in vitro and in vivo [64,65]. Transferrin receptor 1 and divalent metal transporter 1 have also been found to be upregulated in breast cancer cells [67]. Decreased FPN in triple negative breast cancer cells has also been found to stimulate cell proliferation and migration likely a result of increase cellular iron levels [68]. Hepcidin promoter DNA has been found to be hyper-methylated in human hepatocellular carcinoma resulting in its transcriptional repression [69]. In prostate cancer, the prostate epithelial cells markedly increase synthesis of hepcidin leading to cancer growth and progression [70]. Overexpression of BMPs 2, 4 and 7 have all been linked to increased hepcidin expression in a number of cancers including prostate [70] and lung [71]. Lastly, TMPRSS6 has been found to be downregulated in triple negative breast cancer [72].

Iron levels have also been suggested to play a role in the progression of Alzheimer’s disease. Rogers et al. discovered that amyloid-beta precursor proteins (AβPP) mRNA contains IREs in the 5’ untranslated region UTR. These IREs interact with IRPs to stabilise the mRNA [73]. In addition, AβPP has been demonstrated to assist in retaining FPN on the cell surface of neurons. However, how AβPP supports FPN retention is still unclear [74]. Altered function of the protein α-synuclein has been demonstrated to play a pivotal role in the pathology of Parkinson’s disease [75]. Increased iron levels within the substantia nigra region of the brain have also been associated with the pathogenesis of Parkinson’s disease [76]. This iron overload is believed to result from the dysregulated expression of ferritin, divalent metal transporter 1 and ferroxidase [75].

An understanding of how hepcidin and FPN are regulated has provided us with candidates which can be tested to target the expression of hepcidin and FPN with the aim to modulate iron levels. An increasing body of evidence suggesting that altered iron levels play a role in disease progression in multiple conditions warrants development of therapeutic strategies to modulate iron levels. One of the ways to do this is to regulate the hepcidin–FPN axis. Several promising candidates have been tested in laboratories, of which several are undergoing clinical trials. The following sections summarise the current status of these approaches.

## 5. Current Therapeutic Treatments for Hepcidin Deficiency

A reduction or loss of hepcidin expression or function leads to increased iron being released into the blood. The current treatment for the iron overload disorder hereditary hemochromatosis (HH) involves weekly phlebotomy as tolerated by the patient (usually totalling no more than 500 mL) until the patient reaches a plasma ferritin level of approximately 50–100 µg/L [77,78]. Once this has been achieved, the goal of the treatment is to maintain these levels. This is normally achieved through additional phlebotomy every three to six months to maintain normal body iron levels [77,78]. However, phlebotomy is not effective for reversing arthropathy, diabetes, cardiomyopathy, cirrhosis and hypogonadism [77,78,79], which are some of the related symptoms/pathologies that develop in hemochromatosis patients.

In addition to phlebotomy, several iron chelators including deferoxamine (DFO), deferiprone (DFP) and deferasirox (DFX) have also been used in the treatment of hereditary hemochromatosis (HH) [80].

## 6. Current Therapeutic Treatments for Lowering Hepcidin

### 6.1. Direct Hepcidin Inhibitors

#### 6.1.1. Anti-Hepcidin Antibodies

Since the discovery of hepcidin as a key player in the regulation of iron homeostasis, it has been the target of multiple drug candidates to prevent it from interacting with FPN. Direct hepcidin inhibition has been investigated as a possible route for normalising hepcidin levels in patients suffering from hepcidin over expression, such as the anaemia of chronic disease (ACD) and iron-refractory iron-deficiency anaemia (IRIDA). Humanised monoclonal antibodies have been developed that display high affinity towards hepcidin leading to its premature degradation. Antibody (Ab) LY2787106 was shown to be well tolerated during its phase one clinical trial, demonstrating a significant increase in serum iron levels. Unfortunately, these increases were only transient and after eight days returned to baseline [81]. Sasu et al. developed the antibody, Ab2.7 which when used in combination with erythropoietin stimulating agents (ESAs) was shown to reverse hepcidin induced anaemia in a heat killed *Brucella abortus* induced mouse model [82]. The combined treatment was also shown to increase reticulocyte numbers. However, when compared with another direct hepcidin inhibitor, short hairpin RNA (shRNA), the antibody delivered a lower level of hepcidin inhibition. This may have been due to the affinity of the antibody or due to the high turnover of hepcidin within mouse models [82].

#### 6.1.2. Short Interfering and Short Hairpin RNA

Short interfering RNA (siRNA) causing *HEPCIDIN* gene silencing represents another area of active development into the treatment of anaemia. Short hairpin RNAs (shRNAs) targeting hepcidin (H6 and H10) developed by Amgen were demonstrated to cause a reduction in hepcidin mRNA and anaemia when used in conjunction with ESAs [82]. As discussed above, these shRNAs displayed a more robust hepcidin inhibition than the anti-hepcidin antibodies (Ab2.7). Either H6 or H10 treated anaemic mice displayed increased serum iron compared to anaemic control mice treated with shRNAs [82]. The siRNA, ALN-HPN has also been shown to decrease hepcidin mRNA (> 80%) and increase serum iron (approximately two-fold) with a single intravenous dose [83].

#### 6.1.3. Hepcidin-Binding Molecules

Anticalins are therapeutic ligand binding proteins developed from lipocalins [84]. Lipocalins are responsible for the transport of hydrophobic and chemically sensitive molecules in the human body [84]. PRS-080 is a human neutrophil gelatinase-associated lipocalin-derived anticalin engineered for hepcidin binding which results in decreased hepcidin protein levels and subsequently increased iron and transferrin saturation [85]. PRS-080 specifically binds human hepcidin with a dissociation constant (K_d_) in the subnanomolar range (0.07 ± 0.05 nM) and cynomolgus monkey hepcidin (0.07 ± 0.06 nM) [86]. To decrease the rate of kidney filtration for PRS-080, multiple (20, 30 and 40 kDa) polyethylene glycol moieties were conjugated to PRS-080, which showed no change in activity compared with unmodified PRS-080, while increasing the half-life in cynomolgus monkey (18.8 ± 1.1, 43.5 ± 6.0 and 167.5 ± 10.6 h, respectively) [86]. PRS-080 conjugated to the 30 kDa PEG (PRS-080#22) has undergone phase one clinical trials [87]. Healthy male patients were treated with IV doses of 0.08–16 mg/kg over a 2-h period. Decreases in serum hepcidin levels were recorded 1-h post infusion, with corresponding increases in serum iron and transferrin saturation from doses 0.4 mg/kg and higher [87]. Prolonged elevated serum iron and transferrin saturation levels were demonstrated to be dose dependent with increases from 18 h (0.4 mg/kg) to 120 h (16 mg/kg) [87]. These promising results have resulted in PRS-080 progressing towards repeat dosage clinical trials. Another phase 1 clinical trial for PRS-080#22 displayed no adverse effects when given to healthy patients (16 mg/kg) and chronic kidney disease (CKD) patients undergoing haemodialysis (8 mg/kg). In addition, serum iron and transferrin saturation were both increased with PRS-808#22 treatment with the authors suggesting this indicates that the iron was transferrin bound and therefore highly functional [88].

In parallel, the organic phosphate guanosine 5,-diphosphate (GDP) has been shown to complex with hepcidin at the active site via multiple stable hydrogen bonds [89]. This complex then prevents hepcidin from interacting with FPN, resulting in reduced internalisation of FPN in HepG2 and Caco-2 cell lines. GDP appears to be unique in this sense, as other organic phosphates do not affect FPN degradation [89].

#### 6.1.4. Hepcidin-Binding L-RNA Aptamers (Spiegelmers)

Aptamers are an emerging class of synthetic, structured oligonucleotide therapeutics. They consist of DNA, RNA or nucleotides ordered around a modified sugar backbone which display high affinity and specificity [90]. Nox-H94, a PEGylated anti-hepcidin L-RNA aptamer, has been shown to minimise hepcidin-induced FPN degradation and ferritin expression in cynomolgus monkeys [91]. A human study involving healthy adults investigated the safety, pharmacokinetics and pharmacodynamics of Nox-H94 and showed no serious adverse health effects [92] while resulting in an increase in serum iron and transferrin saturation levels [92] (Table 1).

### 6.2. Inhibitors of Hepcidin Production/Synthesis

One of the most important pathways involved in regulating systemic hepcidin levels in response to several stimuli is the BMP-SMAD pathway [131]. Several studies have focussed on targeting this pathway to alter hepcidin levels and normalise iron levels. This section summarises a number of these approaches.

#### 6.2.1. Heparin-Based Targeting of the BMP/SMAD Pathway

Heparin is comprised of repeating units of uronic acid and D-glucosamine or D-glucosamine N-sulphate. Unfractionated heparin (>4 µg/mL) was shown to sequester BMPs and block SMAD phosphorylation, which then resulted in reduced hepcidin mRNA in HepG2 cells [93]. This hepcidin lowering effect was also found with low molecular weight heparin, enoxaparin and fondaparinux [93]. These types of heparins require significantly greater concentrations (40 µg/mL and > 200 µg/mL, respectively) to repress hepcidin levels. However, the anticoagulant effect of heparin makes it difficult to use in mouse models [94]. Glycol-split variants, RO-68 and RO-82 have both been shown to lack anti-thrombin binding while remaining potent hepcidin inhibitors [94]. Super-sulphated heparin SSLMWH-19 was demonstrated to exhibit an even greater degree of hepcidin inhibition than either RO-68 or RO-82. However, SSLMWH-19 still retained marginal anticoagulant properties [95].

#### 6.2.2. Bone Morphogenetic Protein Receptor (BMPR) Inhibitors

Dorsomorphin has been shown to inhibit type 1 BMPRs, which in turn results in decreased SMAD1/5/8 phosphorylation in zebrafish and mouse models [96]. LDN-193189, a dorsomorphin derivative, has been demonstrated to inhibit BMP4-mediated SMAD1/5/8 phosphorylation in rats with ACD, followed by an increase in serum iron and FPN levels and decrease in ferritin levels in the spleen [97]. Asshoff et al. recently developed the antibody momelotinib that targets ALK2 leading to a reduction in hepcidin production in anaemic rats [98]. Lastly, TP-0184 demonstrated an inhibitory effect against ALK2 with two oral doses eight hours apart ameliorating turpentine oil mediated anaemia in mice [99]. While both momelotinib and TP-0184 appear to be specific for only ALK2, both dorsomorphin and LDN-1913189 have been found to have off target effects, which reduces their potential as a therapeutic agents [132].

Two clinically approved drugs (imatinib and spironolactone) were identified that decrease hepcidin through the BMP6 pathway in a variety of cell types (HuH7 and primary hepatocytes of both human and mouse origin) and in male wild-type C57BL/6 mice [100]. Unfortunately, imatinib is not a suitable target for drug re-purposing due to several adverse effects including fatigue, nausea, vomiting, rash, peripheral oedema and abdominal pain. In contrast, spironolactone displays generally minor side effects [100].

#### 6.2.3. Hemojuvelin (HJV) and Transferrin Receptor 2 (TFR2) Inhibitors

The BMP co-receptor HJV has been a major focus for hepcidin inhibition with a variety of techniques developed. Soluble HJV (sHJV) has previously been implicated in hepcidin inhibition [27]. sHJV fused with immunoglobulin fragment crystallisable region (sHJV.Fc) also displays hepcidin inhibitory ability via decreased SMAD1/5/8 phosphorylation. Treatment of ACD in rats with sHJV.Fc significantly increased haemoglobin levels after a 21-day period of *Streptococcal peptidoglycan-polysaccharide* treatment [97]. Boser et al. created HJV targeting antibodies (ABT-207 or h5F9-AM8) that after a single dose have been shown to increase serum iron and decrease unsaturated iron binding capacity (UIBC) in a rat model [101].

Several studies in humans and mice have shown that TFR2 is required for the proper regulation of hepcidin [133,134,135]. TFR2 also plays a role in the interaction of erythropoietin (EPO) and its receptor (EPO-R) [136]. TRF2 is required for the transport of the EPO-R from the endoplasmic reticulum to the cells surface [137]. Therefore, therapeutics have been developed that target the expression TFR2 with the aim of reducing hepcidin expression. A single IV dose of RNAi against TFR2 in mice led to a marked decrease in TFR2 and hepcidin mRNA while concurrently increasing transferrin saturation [83].

#### 6.2.4. Targeting the IL-6/STAT3 Pathway

IL-6-mediated JAK-STAT activation of hepcidin is another major pathway involved in hepcidin regulation. Hence, blocking the IL-6 pathway has also been investigated as a therapeutic treatment for ACD. IL-6 targeting chimeric antibody siltuximab demonstrated decreased hepcidin levels in 97% of multiple myeloma and Castleman disease patients, with 75% of these patients showing haemoglobin increases of > 1.5 g/dL [107]. Another IL-6 targeting antibody, tocilizumab, decreased IL-6 mediated serum hepcidin in multicentric Castleman disease with hepcidin levels falling to within the normal limit in a two-week period [108].

The small molecule AG490 has been shown to be an inhibitor of JAK2 [138]. After a single injection of AG490, a 37% reduction in hepcidin levels was evident after 24 h in an ACD mouse model [102]. The synthetic STAT3 inhibitor phosphopeptide PpYLKTK, which affects STAT dimerisation [103], decreased hepcidin mRNA expression by 35% 2 h post infection in a mouse derived hepatocyte model [103].

AMP-activated protein kinase (AMPK) has recently been demonstrated to be involved in the regulation of hepcidin through inhibition of STAT3 [104]. Wang et al. subcutaneously injected C57BL/6 mice with 100 µL/20 g body weight turpentine for four weeks to induce anaemia of chronic inflammation [104]. Mice were injected with 250 mg/kg metformin (an AMPK activator) displayed increased serum iron and transferrin saturation while the serum hepcidin, Jak2 and phospho-Stat3 levels were reduced [104]. In addition, pre-treatment of C57BL/6 mice with metformin before treatment with IL-6 (3 µL/20 g body weight) reduced hepcidin mRNA and protein levels and restored serum iron levels.

In a retrospective analysis of 83 Chinese type two diabetes mellitus patients, metformin therapy was associated with a decrease in serum hepcidin levels in men [104]. Previously, Fukuda et al. found that intraperitoneal administration of indazole lowered hepcidin levels in mice [105]. However, this molecule had no effect when administered orally [105]. Using this molecule as a base, a specific oral inhibitor of hepcidin, DS79182026, was generated [106]. This molecule has been shown to display low off target kinase inhibition and a dose of 30 mg/kg in an acute inflammatory mouse model significantly reduced serum hepcidin levels [106].

#### 6.2.5. Hypoxia-Inducible Factors (HIF) Stabilisers

HIF stabilisers are another class of drugs initially designed to increase EPO levels in anaemia patients; however, they were also found to indirectly reduce hepcidin levels. These stabilisers act by inhibiting prolyl hydroxylase, which is responsible for degrading the alpha subunits of the HIF complex during normoxia [139].

Pergola et al. are currently developing an oral hypoxia-inducible factor prolyl hydroxylase inhibitor called vadadustat for use in the treatment of anaemia in CKD patients [109]. In a 20-week, double-blinded, randomised, placebo-controlled phase 2B clinical trial, vadaustat was found to significantly increase haemoglobin with decreases in hepcidin levels by six [109]. Another HIF stabiliser, roxadustat developed by Besarab et al. underwent a proof of concept phase 2B trial in 2015 on newly initiated dialysis patients who previously had not taken EPO analogues [110]. After four-week roxadustat treatment, hepcidin levels significantly decreased in all patient cohorts [110]. Recently, roxadustat has undergone two phase three trials in China undertaken with CDK patients who displayed an increase in haemoglobin levels above those of the controls [140,141]. Lastly, daproustat a competitive reversible inhibitor of PHDs through its interactions with the catalytic iron and subsequent blocking of substrate entry [142] has also been tested as a hepcidin targeting molecule. In a phase 2A randomised trial of stage 3–5 CKD patients, a decrease in hepcidin and subsequent increase in total iron levels in the daprodustat treated cohort was observed [111].

## 7. Current Therapeutic Treatments Targeting Ferroportin

### Ferroportin Agonists

Instead of inhibiting the expression of hepcidin, preventing FPN degradation has also been investigated as a therapeutic treatment for high hepcidin disorders. A high-throughput screen discovered that a thiamine derivative, fursultiamine, inhibited hepcidin binding to FPN, thus stabilising iron export in the HEK293-FPN-GFP cell line [112]. Unfortunately, in vivo assessment of fursultiamine showed no effect on FPN levels, as fursultiamine is a therapeutic replacement for thiamine and is thus rapidly metabolised within the body. However, the metabolite thiamine was found not to attenuate FPN internalisation [112].

In 2012, Eli Lilly filed a patent for an anti-FPN mouse antibody that inhibits hepcidin-mediated internalisation resulting in maintained FPN function [113]. However, to our knowledge, no further progress has been made with this antibody.

Ross et al. described anti-hepcidin antibodies (38G6 and 38C8) that when pre-incubated with Hek-RExTMFPN-V5/BLA cells demonstrated a marginal ability to inhibit hepcidin induced internalisation of FPN [143]. A small molecule screen conducted by Ross et al. also discovered that sulfonyl can inhibit RhoG-hepcidin at concentrations of 141 nM by interacting with cysteine 326 [143]. Further, Sulfonyl quinoxaline has been shown to form an irreversible complex with FPN and demonstrates some affinity of towards the mature FPN [143].

## 8. Current Therapeutic Treatments for Increasing Hepcidin

### 8.1. Hepcidin Agonists

Minihepcidins containing the first 7–9 N-terminal amino acids of hepcidin have been shown to function using a similar mechanism to full length hepcidin, reducing FPN and iron levels in mouse livers [144]. Minihepcidin molecules are rationally designed peptides generated via mutagenesis of both the hepcidin amino acid sequence discussed above and ferroportin’s hepcidin binding motif [144]. The minihepcidin, PR73 caused hypoferremia and increased the survival of *Hepcidin^-/-^* mice when infected with *Vibrio vulnificus*. In contrast, a significant number of untreated *Hepcidin^-/-^* mice died due to infection [117]. Similarly, a single dose of PR65 in iron overloaded *Hepcidin^-/-^* mice resulted in an 85% reduction in serum iron levels after a 24 h period, with iron levels returning to baseline after 48 h [118].

In an attempt to reduce the cost of generating minihepcidins resulting from the use of non-natural amino acids, Chua et al. developed a series of cyclic minihepcidins with mHS17 and mHS26 displaying the highest ferroportin binding affinity [119]. While the cost to manufacture these cyclic minihepcidins is significantly lower than PR73 (67% and 76% decrease in cost, respectively), both displayed a 10-fold decrease in half maximal effective concentration (EC_50_) when compared to PR73 in vitro [119]. In addition, in vivo neither mHS17 nor mHS26 displayed any effect on serum iron levels [119].

Casu et al. recently developed two minihepcidins molecules, M004 and M009 [120]. M004 was shown to decrease FPN expression and serum iron over a 24-h period. However, it was discontinued due to the higher stability of M009. In a β-thalassaemia mouse model, *Hbb^(th3/+)^* mice, treatment with low dose M009 resulted in decreased transferrin saturation and normalised red blood cell counts while high dose M009 resulted in a worsening of anaemia [120]. In a phase one study another mini-hepcidin, LJPC-401 resulted in a dose-dependent reduction in serum iron in 15 patients with iron overload [129].

Therapeutic targeting of other molecules involved in the downregulation of hepcidin production has also been tried. TMPRSS6 reduces hepcidin expression via reduced membrane HJV. Inhibiting TMPRSS6 expression has thus been an active area of research. RNAi containing lipid nanoparticles (LNP) targeting TMPRSS6 have been shown to decrease TMPRSS6 mRNA while simultaneously increasing hepcidin expression [130]. Treatment with two anti-sense oligonucleotides (ASO) (TMPRSS6-ASO#1 and TMPRSS6-ASO#2) resulted in a 90% reduction in *TMPRSS6* mRNA with a subsequent 4–5-fold increase in hepcidin mRNA expression [145]. The effectiveness of TMPRSS6-ASOs were further validated in a 2015 study by Gou et al., where they found 30% reduction in transferrin saturation and 40–50% reduction in liver iron levels in a β-thalassemia mouse model [121]. In addition, sustained dosing of TMPRSS6-ASO in a monkey model reduced serum iron levels [121]. Aghajan et al. developed TMPRSS6-ASO conjugated to triantennary N-acetyl galactosamine (GalNAc). When compared with the unconjugated ASO, the GalNAc-ASO demonstrated a 10-fold improvement in EC_50_ and resulted in a similar decrease in serum iron [122]. Recently, Schmidt et al. conjugated siRNA conjugated to GalNAc, which resulted in a two-fold increase in hepcidin expression in splenectomised *Hbb^(th3/+)^* mice [123].

Based upon the success of TMPRSS6-ASOs, Casu et al. performed a six-week study on *Hbb^(th3/+)^* mice treated with TMPRSS6-ASOs or TMPRSS6-ASOs in combination with deferiprone (DFP) [146]. As expected, administration of TMPRSS6-ASO either alone or with DFP increased hepcidin levels, while DFP alone did not. TMPRSS6-ASO and DFP treatment displayed a synergistic effect on liver iron content [146]. The success of inhibiting TMPRSS6 with concomitant chelator treatment was also shown by Schimdt et al. using TMPRSS6 RNAi and DFP to reduce secondary iron overload in *Hbb^(th3/+)^* mice [147]. These positive results have formed the basis for a clinical trial that commenced in 2017 [122].

### 8.2. Hepcidin Analogues

Mature hepcidin contains eight highly conserved disulphide bonds at positions cysteine (Cys) seven and Cys23, Cys10 and Cys13, Cys11 and Cys19, and Cys14 and Cys22 [114]. Recently, Pandur et al. created a hepcidin analogue that displayed similar FPN binding and ubiquitination characteristics as wild type (WT) hepcidin [114]. Using site directed mutagenesis, Pandur et al. probed the effect of replacing each cysteine residue with serine residues on the binding and activity of the mature peptide. They revealed that cysteine 19 is not essential, as the mutation still retained full biologic activity [114]. In addition, intracellular iron levels increased within cells treated with either WT hepcidin or the Cys19 mutant, a result not seen in any other cysteine mutants. Lastly, Pandur et al. demonstrated using ELISA that over a 96-h period the cys19ser mutant hepcidin remained in a higher active concentration when incubated in media compared to WT hepcidin [114]. As mentioned previously minihepcidins are costly to produce due to unnatural amino acids and C-terminal lipid tails. Thus, the lack of unnatural amino acids and C-terminal lipid tail should make this hepcidin mutant a promising therapeutic candidate.

### 8.3. Small Molecule Hepcidin Agonists

Several phytoestrogens (naringenin, quercetin and resveratrol) found in fruits and vegetables have been shown to increase *hepcidin* expression through interactions with Nrf2 and an antioxidant response element located within the hepcidin promoter within a rat model [124]. HepG2 cells treated for six hours with naringenin, quercetin and resveratrol displayed an increase in *hepcidin* expression of approximately 2.5-, 3.5- and 3.5-fold, respectively [124]. In rats treated with quercetin, *hepcidin* expression was increased by 500-fold, wWhile naringenin and resveratrol increased *hepcidin* expression approximately 4- and 12.5-fold, respectively [124].

Mleczko-Sanecka et al. employed siRNA and small molecule screens, which highlighted the role of Ras GTPase (RAS)/RAF proto-oncogene serine/threonine-protein kinase (RAF) and phosphoinositide-3 kinase (PI3K) pathway inhibitors (sorafenib, wortmannin and rapamycin) as inducers of hepcidin mRNA in Hep3B cells [115]. Another screen of 22 water-soluble and fat-soluble vitamins conducted by Zhang et al. identified adenine (vitamin B4) as a potent activator of hepcidin which acts through the BMP6/SMAD pathway [125]. HUH7 cells treated with 50 µM adenine for 12 h displayed an increase of 4.6-fold in hepcidin expression. When iron loaded C57/BL6 mice were fed a diet containing 0.2% (*w/w*) adenine for up to 10 days, hepcidin mRNA expression was increased with subsequent reductions in serum iron and liver iron and transferrin saturation [125].

Recently, a Kuntiz-type hepatocyte growth factor activator inhibitor two (HAI-2) that targets and inhibits the proteolytic activity of TMPRSS6 was discovered [148]. HAI-2 prevented HJV cleavage by forming a series of hydrogen bonds and a disulphide bond within the TMPRSS6 active site which prevents hepcidin downregulation [148].

Zhen et al. found that the isoflavone, genistein was able to increase hepcidin expression levels in zebrafish embryos, while the inactive form of genistein, genistin and other related compounds diazdein (isoflavone) and apigenin (flavone) had no effect on hepcidin transcription [126]. This hepcidin induction requires both BMP/SMAD and JAK/STAT3 pathways as BMP-RE and STAT binding motifs are critical for proper genistein activity [126]. However, genistiein appears to also act through BMP-RE independent mechanisms as HepG2 cells treated with both dorsmorphin and genistein displayed increased hepcidin expression levels over dorsomorphin treated cells alone. Unfortunately, genistein was found to inhibit cell proliferation and increase STAT3 phosphorylation in high concentrations (25–100 µM). It also increases apoptosis at 200 µM [149]. In addition, Chau et al. found that transgenic adenocarcinoma mouse prostate (TRAMP) mice treated with 250 mg/kg of genistein had a three-fold increase in prostate weight due to increases in telomerase activity [150].

Gaun, V. et al. employed a small molecule library screen to determine modulators of hepcidin expression. This screen of over 10,000 molecules using HepG2 cells expressing the luciferase gene under the control of the *hepcidin* promoter, identified 20 agonists and one antagonist [127]. The activity of these 21 molecules was then screened using real time PCR. Sixteen of the identified agonists increased both luciferase and endogenous hepcidin expression [127]. Meanwhile, the lone antagonist increased hepcidin transcription levels despite decreasing hepcidin-luciferase activity. The majority of chemicals identified (six) were shown to increase hepcidin expression through the BMP6 pathway, while only four chemicals increased hepcidin expression through the STAT3 pathway and the remaining six chemicals were found to work through both the BMP6 and STAT3 pathway [127]. Of note, ipriflavone and vorinostat were found to elicit an effect on hepcidin expression at concentrations 10-fold lower than those required for genistein [127]. Due to the higher potency of ipriflavone, C57BL/6 male mice were treated with increasing concentrations of ipriflavone for 50 days to determine changes in iron content, hepcidin and ferroportin expression levels [151]. The treated mice displayed a significant reduction in liver iron content of approximately 40% compared to control untreated mice. Ipriflavone also resulted in an approximate two-fold increase in hepcidin mRNA levels [151]. The hepcidin inducing effect of vorinstat was further confirmed in another study using HUH7 and primary hepatocytes from both human and mice origin [100]. Unfortunately, these effects were not seen in a C57BL/6 mouse model [100]. Another high-throughput screen conducted in zebrafish by Li et al. discovered three steroid molecules, namely epitiostanol, progesterone, and mifepristone, which increase hepcidin expression [116]. These molecules were found to activate hepcidin synthesis through the progesterone receptor membrane component 1 (PGRMC1) [116]. Zhang et al. in 2016 employed a natural product screen using traditional Chinese herbal medicinal plants that identified icariin as an inducer of hepcidin expression in HepG2 and Hepa 1–6 cells as well as in wild-type ICR mice [128]. Icariin was found to interact with the hepcidin regulatory pathway through increased phosphorylation of both STAT3 and Smad1/5/8 [128]. Similar results were seen for icariin analogues epimedin A, B and C when administered to mice, with epimedin C demonstrating the most significant increase in expression. A second compound berberine was also found to increase hepcidin expression in cell lines. However, berberine failed to increase hepcidin expression in vivo [128].

## 9. Conclusion

The important biological role iron plays in health and disease is exemplified by the myriad of therapeutics currently under development for the regulation of hepcidin and ferroportin. Most promisingly, some of these drug candidates are currently undergoing clinical trials, e.g., roxadustat, which has recently completed phase three trials. However, many of these developing treatments must still overcome significant challenges before they can be used as therapeutic agents. For instance, RNAi technologies still require improvements in design to avoid off-target interactions, increased stability to enable an appropriate half-life for action and a compatible delivery system for proper localisation within the body [152]. In addition, many of the aforementioned SMAD and STAT therapeutics lack the specificity to target only the pathways involved with hepcidin regulation [2,100]. Continued research into the molecular mechanism of the hepcidin/ferroportin axis will likely provide additional targets which may overcome the abovementioned limitations of the current lot of in development therapeutic treatments.

## Figures and Tables

**Table 1:** Summary table of hepcidin and ferroportin agonists and antagonists.

Model	Drug	Target
**Direct Hepcidin Inhibitors**		
In vitro	Guanosine 5, -diphosphate (GDP) [89]	Hepcidin
In vivo	Ab2.7, H6 and H10 [82], ALN-HPN [83]	
Clinical trial	LY2787106 [81], PRS-080 [87], Nox-H94 [92]	
**Inhibitors of Hepcidin Production/Synthesis**		
In vitro	Unfractionated heparin	BMP6
	Enoxaparin, Fondaparinux [93]	
	RO-68 and RO-82 [94]	
	SSLMWH-19 [95]	
In vivo	Dorsomorphin [96], LDN-1913189 [97]	Type 1 BMPRs
	Momelotinib [98], TP-0184 [99]	ALK2
	Imatinib, spironolactone [100]	BMP/SMAD Pathway
	sHJV.Fc [97]	BMP6
	ABT-207, h5F9-AM8 [101]	HJV
	RNAi [83]	TFR2
	AG490 [102]	JAK
	PpYLKTK [103]	STAT3
	Metformin [104]	AMPK
	Indazole [105], DS79182026 [106]	ALK2 and ALK3
Clinical Trail	Siltuximab [107], Tocilizumab [108]	IL-6
**Hypoxia-Inducible Factors (HIF) Stabilisers**		
Clinical trial	Vadadustat [109], Roxadustat [110], Daproustat [111]	PHD
**Ferroportin Agonists**		
In vitro	Fursultiamine [112], Anti-FPN mouse antibody [113]	FPN
**Hepcidin Agonists**		
In vitro	Hepcidin Cys19Ser [114]	FPN
	Sorafenib, Wortmannin, Rapamycin [115]	RAS/RAF and PI3
	Epitiostanol, Progesterone, Mifepristone [116]	PGRMC1
In vivo	PR73 [117], PR65 [118], mHS17, mHS26 [119], M004, M009 [120]	FPN
	TMPRSS6-ASO#1 [121], TMPRSS6-ASO#2 [121], GalNAc-ASO [122], RNAi-GalNAc [123],	TMPRSS6
	Naringenin, Quercetin, Resveratrol [124]	NRF2
	Adenine [125]	BMP6/SMAD pathway
	Genistein [126]	BMP-RE and STAT
	Ipriflavone, Vorinostat [127]	BMP6 and STAT3
	Icariin, Epimedin [128]	STAT3 and Smad1/5/8
Clinical Trial	LJPC-401 [129]	FPN
	RNAi LNP [130]	TMPRSS6

## References

[1] Liu J., Sun B., Yin H., Liu S. (2016). Hepcidin: A promising therapeutic target for iron disorders: A systematic review. Medicine.

[2] Sebastiani G., Wilkinson N., Pantopoulos K. (2016). Pharmacological targeting of the hepcidin/ferroportin axis. Front. Pharmacol..

[3] Papanikolaou G., Pantopoulos K. (2017). Systemic iron homeostasis and erythropoiesis. IUBMB Life.

[4] Papanikolaou G., Pantopoulos K. (2005). Iron metabolism and toxicity. Toxicol. Appl. Pharmacol..

[5] Muckenthaler M.U., Rivella S., Hentze M.W., Galy B. (2017). A red carpet for iron metabolism. Cell.

[6] Drakesmith H., Nemeth E., Ganz T. (2015). Ironing out ferroportin. Cell Metab..

[7] Park C.H., Valore E.V., Waring A.J., Ganz T. (2001). Hepcidin, a urinary antimicrobial peptide synthesized in the liver. J. Biol. Chem..

[8] Nemeth E., Valore E.V., Territo M., Schiller G., Lichtenstein A., Ganz T. (2003). Hepcidin, a putative mediator of anemia of inflammation, is a type II acute-phase protein. Blood.

[9] Krause A., Sillard R., Kleemeier B., Kluver E., Maronde E., Conejo-Garcia J.R., Forssmann W.G., Schulz-Knappe P., Nehls M.C., Wattler F. (2003). Isolation and biochemical characterization of LEAP-2, a novel blood peptide expressed in the liver. Protein Sci..

[10] Valore E.V., Ganz T. (2008). Posttranslational processing of hepcidin in human hepatocytes is mediated by the prohormone convertase furin. Blood Cells Mol. Dis..

[11] Nemeth E., Preza G.C., Jung C.L., Kaplan J., Waring A.J., Ganz T. (2006). The N-terminus of hepcidin is essential for its interaction with ferroportin: Structure-function study. Blood.

[12] Ganz T. (2013). Systemic iron homeostasis. Physiol. Rev..

[13] Canali S., Zumbrennen-Bullough K.B., Core A.B., Wang C.Y., Nairz M., Bouley R., Swirski F.K., Babitt J.L. (2017). Endothelial cells produce bone morphogenetic protein 6 required for iron homeostasis in mice. Blood.

[14] Andriopoulos B., Corradini E., Xia Y., Faasse S.A., Chen S.Z., Grgurevic L., Knutson M.D., Pietrangelo A., Vukicevic S., Lin H.Y. (2009). BMP6 is a key endogenous regulator of hepcidin expression and iron metabolism. Nat. Genet..

[15] Meynard D., Kautz L., Darnaud V., Canonne-Hergaux F., Coppin H., Roth M.P. (2009). Lack of the bone morphogenetic protein BMP6 induces massive iron overload. Nat. Genet..

[16] Truksa J., Peng H., Lee P., Beutler E. (2006). Bone morphogenetic proteins 2, 4, and 9 stimulate murine hepcidin 1 expression independently of Hfe, transferrin receptor 2 (Tfr2), and IL-6. Proc. Natl. Acad. Sci. USA.

[17] Canali S., Wang C.-Y., Zumbrennen-Bullough K.B., Bayer A., Babitt J.L. (2017). Bone morphogenetic protein 2 controls iron homeostasis in mice independent of Bmp6. Am. J. Hematol..

[18] Babitt J.L., Huang F.W., Wrighting D.M., Xia Y., Sidis Y., Samad T.A., Campagna J.A., Chung R.T., Schneyer A.L., Woolf C.J. (2006). Bone morphogenetic protein signaling by hemojuvelin regulates hepcidin expression. Nat. Genet..

[19] Steinbicker A.U., Bartnikas T.B., Lohmeyer L.K., Leyton P., Mayeur C., Kao S.M., Pappas A.E., Peterson R.T., Bloch D.B., Yu P.B. (2011). Perturbation of hepcidin expression by BMP type I receptor deletion induces iron overload in mice. Blood.

[20] Mayeur C., Leyton P.A., Kolodziej S.A., Yu B.L., Bloch K.D. (2014). BMP type II receptors have redundant roles in the regulation of hepatic hepcidin gene expression and iron metabolism. Blood.

[21] Miyazono K., Kamiya Y., Morikawa M. (2010). Bone morphogenetic protein receptors and signal transduction. J. Biochem..

[22] Wang R.H., Li C., Xu X., Zheng Y., Xiao C., Zerfas P., Cooperman S., Eckhaus M., Rouault T., Mishra L. (2005). A role of SMAD4 in iron metabolism through the positive regulation of hepcidin expression. Cell Metab..

[23] Truksa J., Lee P., Beutler E. (2009). Two BMP responsive elements, STAT, and bZIP/HNF4/COUP motifs of the hepcidin promoter are critical for BMP, SMAD1, and HJV responsiveness. Blood.

[24] An P., Wang H., Wu Q., Wang J., Xia Z., He X., Wang X., Chen Y., Min J., Wang F. (2018). Smad7 deficiency decreases iron and haemoglobin through hepcidin up-regulation by multilayer compensatory mechanisms. J. Cell. Mol. Med..

[25] Meynard D., Vaja V., Sun C.C., Corradini E., Chen S., Lopez-Otin C., Grgurevic L., Hong C.C., Stirnberg M., Gutschow M. (2011). Regulation of TMPRSS6 by BMP6 and iron in human cells and mice. Blood.

[26] Silvestri L., Pagani A., Nai A., De Domenico I., Kaplan J., Camaschella C. (2008). The serine protease matriptase-2 (TMPRSS6) inhibits hepcidin activation by cleaving membrane hemojuvelin. Cell Metab..

[27] Lin L., Goldberg Y.P., Ganz T. (2005). Competitive regulation of hepcidin mRNA by soluble and cell-associated hemojuvelin. Blood.

[28] Verga Falzacappa M.V., Vujic Spasic M., Kessler R., Stolte J., Hentze M.W., Muckenthaler M.U. (2007). STAT3 mediates hepatic hepcidin expression and its inflammatory stimulation. Blood.

[29] Ganz T., Nemeth E. (2015). Iron homeostasis in host defence and inflammation. Nat. Rev. Immunol..

[30] Canali S., Core A.B., Zumbrennen-Bullough K.B., Merkulova M., Wang C.Y., Schneyer A.L., Pietrangelo A., Babitt J.L. (2016). Activin B induces noncanonical SMAD1/5/8 signaling via BMP type I receptors in hepatocytes: Evidence for a role in hepcidin induction by inflammation in male mice. Endocrinology.

[31] Kanamori Y., Sugiyama M., Hashimoto O., Murakami M., Matsui T., Funaba M. (2016). Regulation of hepcidin expression by inflammation-induced activin B. Sci. Rep..

[32] Kaelin W.G., Ratcliffe P.J. (2008). Oxygen sensing by metazoans: The central role of the HIF hydroxylase pathway. Mol. Cell.

[33] Kapitsinou P.P., Liu Q., Unger T.L., Rha J., Davidoff O., Keith B., Epstein J.A., Moores S.L., Erickson-Miller C.L., Haase V.H. (2010). Hepatic HIF-2 regulates erythropoietic responses to hypoxia in renal anemia. Blood.

[34] Morita M., Ohneda O., Yamashita T., Takahashi S., Suzuki N., Nakajima O., Kawauchi S., Ema M., Shibahara S., Udono T. (2003). HLF/HIF-2alpha is a key factor in retinopathy of prematurity in association with erythropoietin. EMBO J..

[35] Liu Q., Davidoff O., Niss K., Haase V.H. (2012). Hypoxia-inducible factor regulates hepcidin via erythropoietin-induced erythropoiesis. J. Clin. Investig..

[36] Peyssonnaux C., Zinkernagel A.S., Schuepbach R.A., Rankin E., Vaulont S., Haase V.H., Nizet V., Johnson R.S. (2007). Regulation of iron homeostasis by the hypoxia-inducible transcription factors (HIFs). J. Clin. Investig..

[37] Volke M., Gale D.P., Maegdefrau U., Schley G., Klanke B., Bosserhoff A.K., Maxwell P.H., Eckardt K.U., Warnecke C. (2009). Evidence for a lack of a direct transcriptional suppression of the iron regulatory peptide hepcidin by hypoxia-inducible factors. PLoS ONE.

[38] Silvestri L., Pagani A., Camaschella C. (2008). Furin-mediated release of soluble hemojuvelin: A new link between hypoxia and iron homeostasis. Blood.

[39] Maurer E., Gutschow M., Stirnberg M. (2012). Matriptase-2 (TMPRSS6) is directly up-regulated by hypoxia inducible factor-1: Identification of a hypoxia-responsive element in the TMPRSS6 promoter region. Biol. Chem..

[40] Lakhal S., Schodel J., Townsend A.R., Pugh C.W., Ratcliffe P.J., Mole D.R. (2011). Regulation of type II transmembrane serine proteinase TMPRSS6 by hypoxia-inducible factors: New link between hypoxia signaling and iron homeostasis. J. Biol. Chem..

[41] Pasricha S.R., McHugh K., Drakesmith H. (2016). Regulation of hepcidin by erythropoiesis: The story so far. Annu. Rev. Nutr..

[42] Tanno T., Bhanu N.V., Oneal P.A., Goh S.H., Staker P., Lee Y.T., Moroney J.W., Reed C.H., Luban N.L., Wang R.H. (2007). High levels of GDF15 in thalassemia suppress expression of the iron regulatory protein hepcidin. Nat. Med..

[43] Tanno T., Porayette P., Sripichai O., Noh S.J., Byrnes C., Bhupatiraju A., Lee Y.T., Goodnough J.B., Harandi O., Ganz T. (2009). Identification of TWSG1 as a second novel erythroid regulator of hepcidin expression in murine and human cells. Blood.

[44] Casanovas G., Vujic Spasic M., Casu C., Rivella S., Strelau J., Unsicker K., Muckenthaler M.U. (2013). The murine growth differentiation factor 15 is not essential for systemic iron homeostasis in phlebotomized mice. Haematologica.

[45] Mirciov C.S., Wilkins S.J., Dunn L.A., Anderson G.J., Frazer D.M. (2017). Characterization of putative erythroid regulators of hepcidin in mouse models of anemia. PLoS ONE.

[46] Kautz L., Jung G., Du X., Gabayan V., Chapman J., Nasoff M., Nemeth E., Ganz T. (2015). Erythroferrone contributes to hepcidin suppression and iron overload in a mouse model of beta-thalassemia. Blood.

[47] Kautz L., Jung G., Valore E.V., Rivella S., Nemeth E., Ganz T. (2014). Identification of erythroferrone as an erythroid regulator of iron metabolism. Nat. Genet..

[48] Daher R., Lefebvre T., Puy H., Karim Z. (2019). Extrahepatic hepcidin production: The intriguing outcomes of recent years. World J. Clin. Cases.

[49] Sow F.B., Florence W.C., Satoskar A.R., Schlesinger L.S., Zwilling B.S., Lafuse W.P. (2007). Expression and localization of hepcidin in macrophages: A role in host defense against tuberculosis. J. Leukoc. Biol..

[50] Schwarz P., Kübler J.A.M., Strnad P., Müller K., Barth T.F.E., Gerloff A., Feick P., Peyssonnaux C., Vaulont S., Adler G. (2012). Hepcidin is localised in gastric parietal cells, regulates acid secretion and is induced by Helicobacter pylori infection. Gut.

[51] Qian Z.M., He X., Liang T., Wu K.C., Yan Y.C., Lu L.N., Yang G., Luo Q.Q., Yung W.H., Ke Y. (2014). Lipopolysaccharides upregulate hepcidin in neuron via microglia and the IL-6/STAT3 signaling pathway. Mol. Neurobiol..

[52] Urrutia P., Aguirre P., Esparza A., Tapia V., Mena N.P., Arredondo M., Gonzalez-Billault C., Nunez M.T. (2013). Inflammation alters the expression of DMT1, FPN1 and hepcidin, and it causes iron accumulation in central nervous system cells. J. Neurochem..

[53] McKie A.T., Marciani P., Rolfs A., Brennan K., Wehr K., Barrow D., Miret S., Bomford A., Peters T.J., Farzaneh F. (2000). A novel duodenal iron-regulated transporter, IREG1, implicated in the basolateral transfer of iron to the circulation. Mol. Cell.

[54] Donovan A., Brownlie A., Zhou Y., Shepard J., Pratt S.J., Moynihan J., Paw B.H., Drejer A., Barut B., Zapata A. (2000). Positional cloning of zebrafish ferroportin1 identifies a conserved vertebrate iron exporter. Nature.

[55] Abboud S., Haile D.J. (2000). A novel mammalian iron-regulated protein involved in intracellular iron metabolism. J. Biol. Chem..

[56] Qiao B., Sugianto P., Fung E., del-Castillo-Rueda A., Moran-Jimenez M.J., Ganz T., Nemeth E. (2012). Hepcidin-induced endocytosis of ferroportin is dependent on ferroportin ubiquitination. Cell Metab..

[57] Marro S., Chiabrando D., Messana E., Stolte J., Turco E., Tolosano E., Muckenthaler M.U. (2010). Heme controls ferroportin1 (FPN1) transcription involving Bach1, Nrf2 and a MARE/ARE sequence motif at position-7007 of the FPN1 promoter. Haematologica.

[58] Sangokoya C., Doss J.F., Chi J.T. (2013). Iron-responsive miR-485-3p regulates cellular iron homeostasis by targeting ferroportin. PLoS Genet..

[59] Galy B., Ferring-Appel D., Becker C., Gretz N., Grone H.J., Schumann K., Hentze M.W. (2013). Iron regulatory proteins control a mucosal block to intestinal iron absorption. Cell Rep..

[60] Nairz M., Ferring-Appel D., Casarrubea D., Sonnweber T., Viatte L., Schroll A., Haschka D., Fang F.C., Hentze M.W., Weiss G. (2015). Iron regulatory proteins mediate host resistance to salmonella infection. Cell Host Microbe.

[61] Agoro R., Mura C. (2016). Inflammation-induced up-regulation of hepcidin and down-regulation of ferroportin transcription are dependent on macrophage polarization. Blood Cells Mol. Dis..

[62] Yang F., Liu X.B., Quinones M., Melby P.C., Ghio A., Haile D.J. (2002). Regulation of reticuloendothelial iron transporter MTP1 (Slc11a3) by inflammation. J. Biol. Chem..

[63] Vela D., Vela-Gaxha Z. (2018). Differential regulation of hepcidin in cancer and non-cancer tissues and its clinical implications. Exp. Mol. Med..

[64] Guo W., Zhang S., Chen Y., Zhang D., Yuan L., Cong H., Liu S. (2015). An important role of the hepcidin-ferroportin signaling in affecting tumor growth and metastasis. Acta Biochim. Biophys. Sin..

[65] Pinnix Z.K., Miller L.D., Wang W., D’Agostino R., Kute T., Willingham M.C., Hatcher H., Tesfay L., Sui G., Di X. (2010). Ferroportin and iron regulation in breast cancer progression and prognosis. Sci. Transl. Med..

[66] Zhang S., Chen Y., Guo W., Yuan L., Zhang D., Xu Y., Nemeth E., Ganz T., Liu S. (2014). Disordered hepcidin-ferroportin signaling promotes breast cancer growth. Cell. Signal..

[67] Jiang X.P., Elliott R.L., Head J.F. (2010). Manipulation of iron transporter genes results in the suppression of human and mouse mammary adenocarcinomas. Anticancer Res..

[68] Shan Z., Wei Z., Shaikh Z.A. (2018). Suppression of ferroportin expression by cadmium stimulates proliferation, EMT, and migration in triple-negative breast cancer cells. Toxicol. Appl. Pharmacol..

[69] Udali S., Guarini P., Ruzzenente A., Ferrarini A., Guglielmi A., Lotto V., Tononi P., Pattini P., Moruzzi S., Campagnaro T. (2015). DNA methylation and gene expression profiles show novel regulatory pathways in hepatocellular carcinoma. Clin. Epigenet..

[70] Tesfay L., Clausen K.A., Kim J.W., Hegde P., Wang X., Miller L.D., Deng Z., Blanchette N., Arvedson T., Miranti C.K. (2015). Hepcidin regulation in prostate and its disruption in prostate cancer. Cancer Res..

[71] Chen Q., Wang L., Ma Y., Wu X., Jin L., Yu F. (2014). Increased hepcidin expression in non-small cell lung cancer tissue and serum is associated with clinical stage. Thorac. Cancer.

[72] Tuhkanen H., Hartikainen J.M., Soini Y., Velasco G., Sironen R., Nykopp T.K., Kataja V., Eskelinen M., Kosma V.M., Mannermaa A. (2013). Matriptase-2 gene (TMPRSS6) variants associate with breast cancer survival, and reduced expression is related to triple-negative breast cancer. Int. J. Cancer.

[73] Rogers J.T., Randall J.D., Cahill C.M., Eder P.S., Huang X., Gunshin H., Leiter L., McPhee J., Sarang S.S., Utsuki T. (2002). An iron-responsive element type II in the 5′-untranslated region of the Alzheimer’s amyloid precursor protein transcript. J. Biol. Chem..

[74] Wong B.X., Tsatsanis A., Lim L.Q., Adlard P.A., Bush A.I., Duce J.A. (2014). Beta-amyloid precursor protein does not possess ferroxidase activity but does stabilize the cell surface ferrous iron exporter ferroportin. PLoS ONE.

[75] Belaidi A.A., Bush A.I. (2016). Iron neurochemistry in Alzheimer’s disease and Parkinson’s disease: Targets for therapeutics. J. Neurochem..

[76] Wang J.Y., Zhuang Q.Q., Zhu L.B., Zhu H., Li T., Li R., Chen S.F., Huang C.P., Zhang X., Zhu J.H. (2016). Meta-analysis of brain iron levels of Parkinson’s disease patients determined by postmortem and MRI measurements. Sci. Rep..

[77] Bacon B.R., Adams P.C., Kowdley K.V., Powell L.W., Tavill A.S., American Association for the Study of Liver Diseases (2011). Diagnosis and management of hemochromatosis: 2011 practice guideline by the American Association for the Study of Liver Diseases. Hepatology.

[78] European Association for the Study of the Liver (2010). EASL clinical practice guidelines for HFE hemochromatosis. J. Hepatol..

[79] Katsarou A., Pantopoulos K. (2018). Hepcidin therapeutics. Pharmaceuticals.

[80] Brissot P. (2016). Optimizing the diagnosis and the treatment of iron overload diseases. Expert Rev. Gastroenterol. Hepatol..

[81] Vadhan-Raj S., Abonour R., Goldman J.W., Smith D.A., Slapak C.A., Ilaria R.L., Tiu R.V., Wang X., Callies S., Cox J. (2017). A first-in-human phase 1 study of a hepcidin monoclonal antibody, LY2787106, in cancer-associated anemia. J. Hematol. Oncol..

[82] Sasu B.J., Cooke K.S., Arvedson T.L., Plewa C., Ellison A.R., Sheng J., Winters A., Juan T., Li H.Y., Begley C.G. (2010). Antihepcidin antibody treatment modulates iron metabolism and is effective in a mouse model of inflammation-induced anemia. Blood.

[83] Alnylam Pharmaceuticals Targeting the Hepcidin Pathway with RNAi Therapeutics for the Treatment of Anemia. http://www.alnylam.com/web/assets/HPN-ALNY-ASH2011-Anemia.pdf.

[84] Schlehuber S., Skerra A. (2005). Lipocalins in drug discovery: From natural ligand-binding proteins to ‘anticalins’. Drug Discov. Today.

[85] Pieris Pharmaceuticals PRS-080: Best-in-Class Hepcidin Antagonist for Anemia. http://www.pieris.com/pipeline/anemia-and-other-disease-areas/prs-080.

[86] Hohlbaum A.M., Gille H., Trentmann S., Kolodziejczyk M., Rattenstetter B., Laarakkers C.M., Katzmann G., Christian H.J., Andersen N., Allersdorfer A. (2018). Sustained plasma hepcidin suppression and iron elevation by Anticalin-derived hepcidin antagonist in cynomolgus monkey. Br. J. Pharmacol..

[87] Moebius U., Feuerer W., Fenzl E., van Swelm R., Swinkels D.W., Hohlbaum A. (2015). A phase I study investigating the safety, tolerability, pharmacokinetics and pharmacodynamic activity of the hepcidin antagonist PRS-080#022. Results from a randomized, placebo controlled, double-blind study following single administration to healthy subjects. Blood.

[88] Renders L., Budde K., Rosenberger C., van Swelm R., Swinkels D., Dellanna F., Feuerer W., Wen M., Erley C., Bader B. (2019). First-in-human phase I studies of PRS-080#22, a hepcidin antagonist, in healthy volunteers and patients with chronic kidney disease undergoing hemodialysis. PLoS ONE.

[89] Angmo S., Tripathi N., Abbat S., Sharma S., Singh S.S., Halder A., Yadav K., Shukla G., Sandhir R., Rishi V. (2017). Identification of guanosine 5′-diphosphate as potential iron mobilizer: Preventing the hepcidin-ferroportin interaction and modulating the interleukin-6/Stat-3 pathway. Sci. Rep..

[90] Pendergrast P.S., Marsh H.N., Grate D., Healy J.M., Stanton M. (2005). Nucleic acid aptamers for target validation and therapeutic applications. J. Biomol. Tech..

[91] Schwoebel F., van Eijk L.T., Zboralski D., Sell S., Buchner K., Maasch C., Purschke W.G., Humphrey M., Zollner S., Eulberg D. (2013). The effects of the anti-hepcidin Spiegelmer NOX-H94 on inflammation-induced anemia in cynomolgus monkeys. Blood.

[92] Boyce M., Warrington S., Cortezi B., Zollner S., Vauleon S., Swinkels D.W., Summo L., Schwoebel F., Riecke K. (2016). Safety, pharmacokinetics and pharmacodynamics of the anti-hepcidin Spiegelmer lexaptepid pegol in healthy subjects. Br. J. Pharmacol..

[93] Poli M., Girelli D., Campostrini N., Maccarinelli F., Finazzi D., Luscieti S., Nai A., Arosio P. (2011). Heparin: A potent inhibitor of hepcidin expression in vitro and in vivo. Blood.

[94] Poli M., Asperti M., Naggi A., Campostrini N., Girelli D., Corbella M., Benzi M., Besson-Fournier C., Coppin H., Maccarinelli F. (2014). Glycol-split nonanticoagulant heparins are inhibitors of hepcidin expression in vitro and in vivo. Blood.

[95] Poli M., Asperti M., Ruzzenenti P., Mandelli L., Campostrini N., Martini G., Di Somma M., Maccarinelli F., Girelli D., Naggi A. (2014). Oversulfated heparins with low anticoagulant activity are strong and fast inhibitors of hepcidin expression in vitro and in vivo. Biochem. Pharmacol..

[96] Yu P.B., Hong C.C., Sachidanandan C., Babitt J.L., Deng D.Y., Hoyng S.A., Lin H.Y., Bloch K.D., Peterson R.T. (2008). Dorsomorphin inhibits BMP signals required for embryogenesis and iron metabolism. Nat. Chem. Biol..

[97] Theurl I., Schroll A., Sonnweber T., Nairz M., Theurl M., Willenbacher W., Eller K., Wolf D., Seifert M., Sun C.C. (2011). Pharmacologic inhibition of hepcidin expression reverses anemia of chronic inflammation in rats. Blood.

[98] Asshoff M., Petzer V., Warr M.R., Haschka D., Tymoszuk P., Demetz E., Seifert M., Posch W., Nairz M., Maciejewski P. (2017). Momelotinib inhibits ACVR1/ALK2, decreases hepcidin production, and ameliorates anemia of chronic disease in rodents. Blood.

[99] Peterson P., Soh K.K., Lee Y.S., Kim W., Whatcott C.J., Siddiqui-Jain A., Bearss D.J., Warner S.L. (2015). ALK2 inhibition via TP-0184 abrogates inflammation-induced hepcidin expression and is a potential therapeutic for anemia of chronic disease. Blood.

[100] Mleczko-Sanecka K., da Silva A.R., Call D., Neves J., Schmeer N., Damm G., Seehofer D., Muckenthaler M.U. (2017). Imatinib and spironolactone suppress hepcidin expression. Haematologica.

[101] Boser P., Seemann D., Liguori M.J., Fan L., Huang L., Hafner M., Popp A., Mueller B.K. (2015). Anti-repulsive guidance molecule C (RGMc) antibodies increases serum iron in rats and cynomolgus monkeys by hepcidin downregulation. AAPS J..

[102] Zhang S.P., Wang Z., Wang L.X., Liu S.J. (2011). AG490: An inhibitor of hepcidin expression in vivo. World J. Gastroenterol..

[103] Turkson J., Ryan D., Kim J.S., Zhang Y., Chen Z., Haura E., Laudano A., Sebti S., Hamilton A.D., Jove R. (2001). Phosphotyrosyl peptides block Stat3-mediated DNA binding activity, gene regulation, and cell transformation. J. Biol. Chem..

[104] Wang M., Xin H., Tang W., Li Y., Zhang Z., Fan L., Miao L., Tan B., Wang X., Zhu Y.Z. (2017). AMPK serves as a therapeutic target against anemia of inflammation. Antioxid. Redox Signal..

[105] Fukuda T., Ueda K., Ishiyama T., Goto R., Muramatsu S., Hashimoto M., Watanabe K., Tanaka N. (2017). Synthesis and SAR studies of 3,6-disubstituted indazole derivatives as potent hepcidin production inhibitors. Bioorg. Med. Chem. Lett..

[106] Fukuda T., Goto R., Kiho T., Ueda K., Muramatsu S., Hashimoto M., Aki A., Watanabe K., Tanaka N. (2017). Discovery of DS79182026: A potent orally active hepcidin production inhibitor. Bioorg. Med. Chem. Lett..

[107] Kurzrock R., Voorhees P.M., Casper C., Furman R.R., Fayad L., Lonial S., Borghaei H., Jagannath S., Sokol L., Usmani S.Z. (2013). A phase I, open-label study of siltuximab, an anti-IL-6 monoclonal antibody, in patients with B-cell non-Hodgkin lymphoma, multiple myeloma, or Castleman disease. Clin. Cancer Res..

[108] Song S.N., Tomosugi N., Kawabata H., Ishikawa T., Nishikawa T., Yoshizaki K. (2010). Down-regulation of hepcidin resulting from long-term treatment with an anti-IL-6 receptor antibody (tocilizumab) improves anemia of inflammation in multicentric Castleman disease. Blood.

[109] Pergola P.E., Spinowitz B.S., Hartman C.S., Maroni B.J., Haase V.H. (2016). Vadadustat, a novel oral HIF stabilizer, provides effective anemia treatment in nondialysis-dependent chronic kidney disease. Kidney Int..

[110] Besarab A., Chernyavskaya E., Motylev I., Shutov E., Kumbar L.M., Gurevich K., Chan D.T., Leong R., Poole L., Zhong M. (2016). Roxadustat (FG-4592): Correction of anemia in incident dialysis patients. J. Am. Soc. Nephrol..

[111] Brigandi R.A., Johnson B., Oei C., Westerman M., Olbina G., de Zoysa J., Roger S.D., Sahay M., Cross N., McMahon L. (2016). A novel hypoxia-inducible factor-prolyl hydroxylase inhibitor (GSK1278863) for anemia in CKD: A 28-day, phase 2A randomized trial. Am. J. Kidney Dis..

[112] Fung E., Sugianto P., Hsu J., Damoiseaux R., Ganz T., Nemeth E. (2013). High-throughput screening of small molecules identifies hepcidin antagonists. Mol. Pharmacol..

[113] Leung D.D.M., Luan P., Manetta J.V., Tang Y., Witcher D.R. (2012). Anti-Ferroportin 1 Monoclonal Antibodies and Uses Thereof. U.S. Patent.

[114] Pandur E., Fekete Z., Tamasi K., Grama L., Varga E., Sipos K. (2018). The C19S substitution enhances the stability of hepcidin while conserving its biological activity. Protein J..

[115] Mleczko-Sanecka K., Roche F., da Silva A.R., Call D., D’Alessio F., Ragab A., Lapinski P.E., Ummanni R., Korf U., Oakes C. (2014). Unbiased RNAi screen for hepcidin regulators links hepcidin suppression to proliferative Ras/RAF and nutrient-dependent mTOR signaling. Blood.

[116] Li X., Rhee D.K., Malhotra R., Mayeur C., Hurst L.A., Ager E., Shelton G., Kramer Y., McCulloh D., Keefe D. (2016). Progesterone receptor membrane component-1 regulates hepcidin biosynthesis. J. Clin. Investig..

[117] Arezes J., Jung G., Gabayan V., Valore E., Ruchala P., Gulig P.A., Ganz T., Nemeth E., Bulut Y. (2015). Hepcidin-induced hypoferremia is a critical host defense mechanism against the siderophilic bacterium vibrio vulnificus. Cell Host Microbe.

[118] Ramos E., Ruchala P., Goodnough J.B., Kautz L., Preza G.C., Nemeth E., Ganz T. (2012). Minihepcidins prevent iron overload in a hepcidin-deficient mouse model of severe hemochromatosis. Blood.

[119] Chua K., Fung E., Micewicz E.D., Ganz T., Nemeth E., Ruchala P. (2015). Small cyclic agonists of iron regulatory hormone hepcidin. Bioorg. Med. Chem. Lett..

[120] Casu C., Oikonomidou P.R., Chen H.Y., Nandi V., Ginzburg Y., Prasad P., Fleming R.E., Shah Y.M., Valore E.V., Nemeth E. (2016). Minihepcidin peptides as disease modifiers in mice affected by beta-thalassemia and polycythemia vera. Blood.

[121] Guo S., Aghajan M., Casu C., Gardenghi S., Booten S., Rivella S., Monia B.P. (2015). Targeting TMPRSS6 using antisense technology for the treatment of beta-thalassemia. Blood.

[122] Aghajan M., Casu C., lo Presti V., Booten S., Monia B.P., Rivella S., Guo S. (2016). Developing a galnac-conjugated tmprss6 antisense therapy for the treatment of β-thalassemia. Blood.

[123] Schmidt P.J., Liu K., Visner G., Fitzgerald K., Fishman S., Racie T., Hettinger J.L., Butler J.S., Fleming M.D. (2018). RNAi-mediated reduction of hepatic Tmprss6 diminishes anemia and secondary iron overload in a splenectomized mouse model of beta-thalassemia intermedia. Am. J. Hematol..

[124] Bayele H.K., Balesaria S., Srai S.K. (2015). Phytoestrogens modulate hepcidin expression by Nrf2: Implications for dietary control of iron absorption. Free Radic. Biol. Med..

[125] Zhang Y., Wang X., Wu Q., Wang H., Zhao L., Wang X., Mu M., Xie E., He X., Shao D. (2018). Adenine alleviates iron overload by cAMP/PKA mediated hepatic hepcidin in mice. J. Cell. Physiol..

[126] Zhen A.W., Nguyen N.H., Gibert Y., Motola S., Buckett P., Wessling-Resnick M., Fraenkel E., Fraenkel P.G. (2013). The small molecule, genistein, increases hepcidin expression in human hepatocytes. Hepatology.

[127] Gaun V., Patchen B., Volovetz J., Zhen A.W., Andreev A., Pollastri M.P., Fraenkel P.G. (2014). A chemical screen identifies small molecules that regulate hepcidin expression. Blood Cells Mol. Dis..

[128] Zhang M., Liu J., Guo W., Liu X., Liu S., Yin H. (2016). Icariin regulates systemic iron metabolism by increasing hepatic hepcidin expression through Stat3 and Smad1/5/8 signaling. Int. J. Mol. Med..

[129] Yaeger D., Piga A., Lal A., Kattamis A., Salman S., Byrnes B., Tidmarsh G., Chawla L. A Phase 1, Placebo-Controlled Study to Determine the Safety, Tolerability, and Pharmacokinetics of Escalating Subcutaneous Doses of LJPC-401 (Synthetic Human Hepcidin) in Healthy Adults. http://lajollapharmaceutical.com/wp-content/uploads/2018/06/EHA_Poster.pdf.

[130] Schmidt P.J., Toudjarska I., Sendamarai A.K., Racie T., Milstein S., Bettencourt B.R., Hettinger J., Bumcrot D., Fleming M.D. (2013). An RNAi therapeutic targeting Tmprss6 decreases iron overload in Hfe(-/-) mice and ameliorates anemia and iron overload in murine beta-thalassemia intermedia. Blood.

[131] Silvestri L., Nai A., Dulja A., Pagani A. (2019). Hepcidin and the BMP-SMAD pathway: An unexpected liaison. Vitam. Horm..

[132] Boergermann J.H., Kopf J., Yu P.B., Knaus P. (2010). Dorsomorphin and LDN-193189 inhibit BMP-mediated Smad, p38 and Akt signalling in C2C12 cells. Int. J. Biochem. Cell Biol..

[133] Wallace D.F., Summerville L., Subramaniam V.N. (2007). Targeted disruption of the hepatic transferrin receptor 2 gene in mice leads to iron overload. Gastroenterology.

[134] Fleming R.E., Feng Q., Britton R.S. (2011). Knockout mouse models of iron homeostasis. Annu. Rev. Nutr..

[135] Camaschella C., Roetto A., Calì A., De Gobbi M., Garozzo G., Carella M., Majorano N., Totaro A., Gasparini P. (2000). The gene TFR2 is mutated in a new type of haemochromatosis mapping to 7q22. Nat. Genet..

[136] Kawabata H. (2019). Transferrin and transferrin receptors update. Free Radic. Biol. Med..

[137] Forejtnikovà H., Vieillevoye M., Zermati Y., Lambert M., Pellegrino R.M., Guihard S., Gaudry M., Camaschella C., Lacombe C., Roetto A. (2010). Transferrin receptor 2 is a component of the erythropoietin receptor complex and is required for efficient erythropoiesis. Blood.

[138] De Vos J., Jourdan M., Tarte K., Jasmin C., Klein B. (2000). JAK2 tyrosine kinase inhibitor tyrphostin AG490 downregulates the mitogen-activated protein kinase (MAPK) and signal transducer and activator of transcription (STAT) pathways and induces apoptosis in myeloma cells. Br. J. Haematol..

[139] Gupta N., Wish J.B. (2017). Hypoxia-inducible factor prolyl hydroxylase inhibitors: A potential new treatment for anemia in patients with CKD. Am. J. Kidney Dis..

[140] Chen N., Hao C., Liu B.C., Lin H., Wang C., Xing C., Liang X., Jiang G., Liu Z., Li X. (2019). Roxadustat treatment for anemia in patients undergoing long-term dialysis. N. Engl. J. Med..

[141] Chen N., Hao C., Peng X., Lin H., Yin A., Hao L., Tao Y., Liang X., Liu Z., Xing C. (2019). Roxadustat for anemia in patients with kidney disease not receiving dialysis. N. Engl. J. Med..

[142] Ariazi J.L., Duffy K.J., Adams D.F., Fitch D.M., Luo L., Pappalardi M., Biju M., DiFilippo E.H., Shaw T., Wiggall K. (2017). Discovery and preclinical characterization of GSK1278863 (daprodustat), a small molecule hypoxia inducible factor-prolyl hydroxylase inhibitor for anemia. J. Pharmacol. Exp. Ther..

[143] Ross S.L., Biswas K., Rottman J., Allen J.R., Long J., Miranda L.P., Winters A., Arvedson T.L. (2017). Identification of antibody and small molecule antagonists of ferroportin-hepcidin interaction. Front. Pharmacol..

[144] Preza G.C., Ruchala P., Pinon R., Ramos E., Qiao B., Peralta M.A., Sharma S., Waring A., Ganz T., Nemeth E. (2011). Minihepcidins are rationally designed small peptides that mimic hepcidin activity in mice and may be useful for the treatment of iron overload. J. Clin. Investig..

[145] Guo S., Casu C., Gardenghi S., Booten S., Aghajan M., Peralta R., Watt A., Freier S., Monia B.P., Rivella S. (2013). Reducing TMPRSS6 ameliorates hemochromatosis and beta-thalassemia in mice. J. Clin. Investig..

[146] Casu C., Aghajan M., Oikonomidou P.R., Guo S., Monia B.P., Rivella S. (2016). Combination of Tmprss6-ASO and the iron chelator deferiprone improves erythropoiesis and reduces iron overload in a mouse model of beta-thalassemia intermedia. Haematologica.

[147] Schmidt P.J., Racie T., Westerman M., Fitzgerald K., Butler J.S., Fleming M.D. (2015). Combination therapy with a Tmprss6 RNAi-therapeutic and the oral iron chelator deferiprone additively diminishes secondary iron overload in a mouse model of beta-thalassemia intermedia. Am. J. Hematol..

[148] Beckmann A.M., Maurer E., Lulsdorff V., Wilms A., Furtmann N., Bajorath J., Gutschow M., Stirnberg M. (2016). En route to new therapeutic options for iron overload diseases: Matriptase-2 as a target for kunitz-type inhibitors. Chembiochem.

[149] Jagadeesh S., Kyo S., Banerjee P.P. (2006). Genistein represses telomerase activity via both transcriptional and posttranslational mechanisms in human prostate cancer cells. Cancer Res..

[150] Chau M.N., El Touny L.H., Jagadeesh S., Banerjee P.P. (2007). Physiologically achievable concentrations of genistein enhance telomerase activity in prostate cancer cells via the activation of STAT3. Carcinogenesis.

[151] Patchen B., Koppe T., Cheng A., Seo Y.A., Wessling-Resnick M., Fraenkel P.G. (2016). Dietary supplementation with ipriflavone decreases hepatic iron stores in wild type mice. Blood Cells Mol. Dis..

[152] Akhtar S., Benter I.F. (2007). Nonviral delivery of synthetic siRNAs in vivo. J. Clin. Investig..

